# Saturation in qualitative research: exploring its conceptualization and operationalization

**DOI:** 10.1007/s11135-017-0574-8

**Published:** 2017-09-14

**Authors:** Benjamin Saunders, Julius Sim, Tom Kingstone, Shula Baker, Jackie Waterfield, Bernadette Bartlam, Heather Burroughs, Clare Jinks

**Affiliations:** 1 Institute for Primary Care and Health Sciences, Keele University, Keele, Staffordshire ST5 5BG UK; 2 School of Health Sciences, Queen Margaret University, Edinburgh, EH21 6UU UK

**Keywords:** Saturation, Data collection, Data analysis, Grounded theory, Qualitative research

## Abstract

Saturation has attained widespread acceptance as a methodological principle in qualitative research. It is commonly taken to indicate that, on the basis of the data that have been collected or analysed hitherto, further data collection and/or analysis are unnecessary. However, there appears to be uncertainty as to how saturation should be conceptualized, and inconsistencies in its use. In this paper, we look to clarify the nature, purposes and uses of saturation, and in doing so add to theoretical debate on the role of saturation across different methodologies. We identify four distinct approaches to saturation, which differ in terms of the extent to which an inductive or a deductive logic is adopted, and the relative emphasis on data collection, data analysis, and theorizing. We explore the purposes saturation might serve in relation to these different approaches, and the implications for how and when saturation will be sought. In examining these issues, we highlight the uncertain logic underlying saturation—as essentially a predictive statement about the unobserved based on the observed, a judgement that, we argue, results in equivocation, and may in part explain the confusion surrounding its use. We conclude that saturation should be operationalized in a way that is consistent with the research question(s), and the theoretical position and analytic framework adopted, but also that there should be some limit to its scope, so as not to risk saturation losing its coherence and potency if its conceptualization and uses are stretched too widely.

## Introduction

In broad terms, saturation is used in qualitative research as a criterion for discontinuing data collection and/or analysis.^1^ Its origins lie in grounded theory (Glaser and Strauss 1967), but in one form or another it now commands acceptance across a range of approaches to qualitative research. Indeed, saturation is often proposed as an essential methodological element within such work. Fusch and Ness (2015: p. 1408) claim categorically that ‘failure to reach saturation has an impact on the quality of the research conducted’;^2^ Morse (2015: p. 587) notes that saturation is ‘the most frequently touted guarantee of qualitative rigor offered by authors’; and Guest et al. (2006: p. 60) refer to it as having become ‘the gold standard by which purposive sample sizes are determined in health science research.’ A number of authors refer to saturation as a ‘rule’ (Denny 2009; Sparkes et al. 2011), or an ‘edict’ (Morse 1995), of qualitative research, and it features in a number of generic quality criteria for qualitative methods (Leininger 1994; Morse et al. 2002).

However, despite having apparently attained something of the status of orthodoxy, saturation is defined within the literature in varying ways—or is sometimes undefined—and raises a number of problematic conceptual and methodological issues (Dey 1999; Bowen 2008; O’Reilly and Parker 2013). Drawing on a number of examples in the literature, this paper seeks to explore some of these issues in relation to three core questions:

- ‘What?’—in what way(s) is saturation defined?
- ‘Where and why?’—in what types of qualitative research, and for what purpose, should saturation be sought?
- ‘When and how?’—at what stage in the research is saturation sought, and how can we assess if it has been achieved?

In addressing these questions, we will explore the implications of different models of saturation—and the theoretical and methodological assumptions that underpin them—for the varying purposes saturation may serve across different qualitative approaches. In doing so, the paper will contribute to the small but growing literature that has critically examined the concept of saturation (e.g. Bowen 2008; O’Reilly and Parker 2013; Walker 2012; Morse 2015; Nelson 2016), aiming to extend the discussion around its conceptualization and use. We will argue not only for greater transparency in the reporting of saturation, as others have done (Bowen 2008; Francis et al. 2010), but also for a more thorough consideration on the part of qualitative researchers regarding how saturation relates to the research question(s) they are addressing, in addition to the theoretical and analytical approach they have adopted, with due recognition of potential inconsistencies and contradictions in its use.

## ‘What?’—in what way(s) is saturation defined?

In their original treatise on grounded theory, Glaser and Strauss (1967: p. 61) defined saturation in these terms:

The criterion for judging when to stop sampling the different groups pertinent to a category is the category’s *theoretical saturation*. *Saturation* means that no additional data are being found whereby the sociologist can develop properties of the category. As he sees similar instances over and over again, the researcher becomes empirically confident that a category is saturated. He goes out of his way to look for groups that stretch diversity of data as far as possible, just to make certain that saturation is based on the widest possible range of data on the category.

Here, the decision to be made relates to further sampling, and the determinant of adequate sampling has to do with the degree of development of a theoretical category in the process of analysis. Saturation is therefore closely related to the notion of theoretical sampling—the idea that sampling is guided by ‘the necessary similarities and contrasts required by the emerging theory’ (Dey 1999: p. 30)—and causes the researcher to ‘combine sampling, data collection and data analysis, rather than treating them as separate stages in a linear process’ (Bryman 2012: p. 18).

Also writing from a grounded theory standpoint, Urquhart (2013: p. 194) defines saturation as: ‘the point in coding when you find that no new codes occur in the data. There are mounting instances of the same codes, but no new ones’, whilst Given (2016: p. 135) considers saturation as the point at which ‘additional data do not lead to any new emergent themes’. A similar position regarding the (non)emergence of new codes or themes has been taken by others (e.g. Birks and Mills 2015; Olshansky 2015).^3^ These definitions show a change of emphasis, and suggest a second model of saturation. Whilst the focus remains at the level of analysis, the decision to be made appears to relate to the emergence of *new* codes or themes, rather than the degree of development of those already identified. Moreover, Urqhart (2013) and Birks and Mills (2015) relate saturation primarily to the termination of analysis, rather than to the collection of new data.

According to Starks and Trinidad (2007: p. 1375), however, theoretical saturation occurs ‘when the complete range of constructs that make up the theory is fully represented by the data’. Whilst not wholly explicit, this definition suggests a third model of saturation with a different directional logic: not ‘given the data, do we have analytical or theoretical adequacy?’, but ‘given the theory, do we have sufficient data to illustrate it?’^4^

If we move outside the grounded theory literature,^5^ a fourth perspective becomes apparent in which there are references to *data* saturation, rather than *theoretical* saturation (e.g. Fusch and Ness 2015).^6^ This view of saturation seems to centre on the question of how much data (usually number of interviews) is needed until nothing new is apparent, or what Sandelowski (2008: p. 875) calls ‘informational redundancy’ (e.g. Francis et al. 2010; Guest et al. 2006). Grady (1998: p. 26) provides a similar description of data saturation as the point at which:

New data tend to be redundant of data already collected. In interviews, when the researcher begins to hear the same comments again and again, data saturation is being reached… It is then time to stop collecting information and to start analysing what has been collected.

Whilst several others have defined data saturation in a similar way (e.g. Hill et al. 2014: p. 2; Middlemiss et al. 2015; Jackson et al. 2015), Legard et al. (2003) seem to adopt a narrower, more individual-oriented perspective on data saturation, whereby saturation operates not at the level of the dataset as a whole, but in relation to the data provided by an individual participant; i.e. it is achieved at a particular point within a specific interview:

Probing needs to continue until the researcher feels they have reached saturation, a full understanding of the participant’s perspective (Legard et al. 2003: p. 152).

From this perspective, the researcher’s response to the data—through which decisions are made about whether or not any new ‘information’ is being generated—is not necessarily perceived as forming part of the analysis itself. Thus, in this model, the process of saturation is located principally at the level of data collection and is thereby separated from a fuller process of data analysis, and hence from theory.

Four different models of saturation seem therefore to exist (Table 1). The first of these, rooted in traditional grounded theory, uses the development of categories and the emerging theory in the analysis process as the criterion for additional data collection, driven by the notion of theoretical sampling; using a term in common use, but with a more specific definitional focus, this model could thus be labelled as *theoretical saturation*. The second model takes a similar approach, but saturation focuses on the identification of new codes or themes, and is based on the number of such codes or themes rather than the completeness of existing theoretical categories. This can be termed *inductive thematic saturation*. In this model, saturation appears confined to the level of analysis; its implication for data collection is at best implicit. In the third model, a reversal of the preceding logic is suggested, whereby data is collected so as to *exemplify* theory, at the level of lower-order codes or themes, rather than to *develop* or *refine* theory. This model can be termed *a priori thematic saturation*, as it points to the idea of pre-determined theoretical categories and leads us away from the inductive logic characteristic of grounded theory. Finally, the fourth model—which, again aligning with the term already in common use, we will refer to as *data saturation*—sees saturation as a matter of identifying redundancy in the data, with no necessary reference to the theory linked to these data; saturation appears to be distinct from formal data analysis.

**Table 1:** Models of saturation and their principal foci in the research process

Model	Description	Principal focus
Theoretical saturation	Relates to the development of theoretical categories; related to grounded theory methodology	Sampling
Inductive thematic saturation	Relates to the emergence of new codes or themes	Analysis
A priori thematic saturation	Relates to the degree to which identified codes or themes are exemplified in the data	Sampling
Data saturation	Relates to the degree to which new data repeat what was expressed in previous data	Data collection

### ‘Hybrid’ forms of saturation

Some authors appear to espouse interpretations of saturation that combine two or more of the models defined above, making its conceptualization less distinct. For example, Goulding (2005) suggests that both data and theory should be saturated within grounded theory, and Drisko (1997: p. 192) defines saturation in terms of ‘the comprehensiveness of both the data collection and analysis’. Similarly, Morse’s view of saturation seems to embody elements of both theoretical and data saturation. She links saturation with the idea of replication, in a way that suggests a process of data saturation:

However, when the domain has been fully sampled – when all data have been collected – then replication of data occurs and, with this replication… the signal of saturation (Morse 1995: p. 148).

Morse notes elsewhere that she is able to tell when her students have achieved saturation, as they begin to talk about the data in more generalized terms and ‘can readily supply examples when asked. These students *know* their data’ (Morse 2015: p. 588). This too suggests a form of data saturation. However, Morse also proposes that saturation is lacking when ‘there are too few examples in each category to identify the characteristics of concepts, and to develop theory’ (Morse 2015: p. 588). This perspective seems to be located firmly in the idea of theory development (as other parts of the quoted papers by Morse make clear), though a heavy emphasis is placed at the level of the data and the way in which the data exemplify theory, thereby seeming to evoke both data and theoretical saturation.

Hennink et al. (2017) go further, appearing to combine elements of all four models of saturation. They firstly identify ‘code saturation’, the point at which ‘no additional issues are identified and the codebook begins to stabilize’ (2017: p. 4), which seems to combine elements of both inductive thematic saturation and data saturation. However, within this approach saturation is discussed as relating not only to codes developed inductively, but also to a priori codes, which echoes the third model: a priori thematic saturation. They go on to distinguish ‘code saturation’ from ‘meaning saturation’; in the latter, the analyst attempts to ‘fully understand conceptual codes or the conceptual dimensions of… concrete codes’ (2017: p. 14). This focus on saturating the dimensions of codes seems more akin to theoretical saturation; however, their analysis remains at the level of codes, rather than theoretical categories developed from these codes, and Hennink et al. explicitly position their approach outside grounded theory methods.

## ‘Where and why?’—in what types of qualitative research, and for what purpose, should saturation be sought?

Morse (2015: p. 587) takes the view that saturation is ‘present in all qualitative research’ and as previously noted, it is commonly considered as the ‘gold standard’ for determining sample size in qualitative research, with little distinction between different types of qualitative research. We question this perspective, and would instead argue—as is suggested by the different models of saturation considered in the previous section—that saturation has differing relevance, and a different meaning, depending on the role of theory, a viewpoint somewhat supported by other commentators who have questioned its application across the spectrum of qualitative methods (Walker 2012; O’Reilly and Parker 2013; van Manen et al. 2016).

In a largely deductive approach (i.e. one that relies wholly or predominantly on applying pre-identified codes, themes or other analytical categories to the data, rather than allowing these to emerge inductively) saturation may refer to the extent to which pre-determined codes or themes are adequately represented in the data—rather like the idea of the categories being sufficiently replete with instances, or ‘examples’, of data, as suggested in the a priori thematic saturation model outlined above. Thus, in their attempt to establish an adequate sample size for saturation, Francis et al. (2010) refer explicitly to research in which conceptual categories have been pre-established through existing theory, and it is significant in this respect that they link saturation with the notion of content validity. In contrast, within a more inductive approach (e.g. the inductive thematic saturation and theoretical saturation models outlined above), saturation suggests the extent to which ‘new’ codes or themes are identified within the data, and/or the extent to which new theoretical insights are gained from the data via this process.

In both the deductive and the inductive approach, we can make sense of the role of saturation, however much it differs in each case, because the underlying approach to analysis is essentially thematic, and usually occurs in the context of interview or focus group studies involving a number of informants. It is less straightforward to identify a role for saturation in qualitative approaches that are based on a biographical or narrative approach to analysis, or that, more generally, include a specific focus on accounts of *individual* informants (e.g. interpretative phenomenological analysis). In such studies, analysis tends to focus more on *strands* within individual accounts rather than on analytical *themes*; these strands are essentially continuous, whereas themes are essentially recurrent. Accordingly, Marshall and Long (2010) suggest that saturation was not appropriate in their study of maternal coping processes, based on narrative methods. Elsewhere, however, a less straightforward picture emerges. Hawkins and Abrams (2007) utilized saturation in the context of a study based on life-history interviews with 39 formerly homeless mentally ill men and women. The authors state: ‘Of the 39 participants, six did not complete a second interview because they were unavailable, impaired, or the research team felt the first interview had achieved saturation’ (p. 2035), suggesting that judgments of saturation were made within each participant’s account. Power et al. (2015) adopted a story-telling approach to women’s experience of post-partum hospitalization, and recruitment continued until data saturation, which was established through ‘the repetition of responses’ (p. 372). Analysis was thematic, and it is not clear whether saturation was determined in relation to themes across participants’ stories, or within individual stories. Similarly, in a study of osteoarthritis in footballers, based on interpretative phenomenological analysis, Turner et al. (2002) employed saturation, which was defined both in terms of the emergence of themes from the analysis and a ‘consensus across views expressed’ (p. 298), which suggests that, notwithstanding the interpretive phenomenological analysis perspective adopted, saturation was sought more across than within cases. Hale et al. (2007: p. 91) argue, however, that saturation is not normally an aim in interpretative phenomenological analysis, owing to the concern to obtain ‘full and rich personal accounts’, which highlights the particular analytical focus within individual accounts in this approach, and van Manen dissociates saturation from phenomenological research more generally (van Manen et al. 2016).

Considering the various types of research in which saturation might feature helps to clarify the purposes it is intended to fulfil. When used in a deductive approach to analysis, saturation serves to demonstrate the extent to which the data instantiate previously determined conceptual categories, whereas in more inductive approaches, and grounded theory in particular, it says something about the adequacy of sampling in relation to theory development (although we have seen that there are differing accounts of how specifically this should be achieved). In narrative research, a role for saturation is harder to discern. Rather than the sufficient development of theory, it might be seen to indicate the ‘completeness’ of a biographical account. However, one could question whether the point at which a participant’s story is interpreted as being ‘complete’—having presumably conveyed everything seen to be relevant to the focus of the study—is, in fact, usefully described by the concept of saturation, given the distance that this moves us away from the operationalization of saturation in broadly thematic approaches. This might, furthermore, lead us to ask whether there is the risk of saturation losing its coherence and utility if its potential conceptualization and uses are stretched too widely.

The same issue is relevant with regard to a number of other, less obvious, purposes that have been proposed for saturation. For example, it has been claimed to demonstrate the trustworthiness of coding (Damschroder et al. 2007)—but as saturation will be a direct and automatic consequence of one’s coding decisions, it is not clear how it can be an independent measure of their quality. Dubé et al. (2016) suggest that saturation says something about (though not conclusively) the ability to extrapolate findings, and Boddy (2016: p. 428) claims that ‘once saturation is reached, the results must be capable of some degree of generalisation’; this seems to move us away from the notion of the theoretical adequacy of an analysis, and the explanatory scope of a theory, toward a much more empirical sense of generalizability. The use of saturation in these two cases could perhaps indicate a degree of confusion in some studies about the meaning of saturation and its purpose, even when taking into account the differing models of saturation outlined earlier. Therefore, we would suggest that for saturation to be conceptually meaningful and practically useful there should be some limit to the purposes to which it can be applied.

## ‘When and how?’—at what stage in the research is saturation sought, and how can we assess if it has been achieved?

### Perspectives taken on saturation

The perspective taken on what is meant by saturation within a given study will have implications for when it will be sought. Taking the fourth model of saturation identified earlier—the data saturation approach, as based on the notion of informational redundancy—it is clear that saturation can be identified at an early stage in the process, as from this perspective saturation is often seen as separate from, and preceding, formal analysis. Decisions about when further data collection is unnecessary are commonly based on the researcher’s sense of what they are hearing within interviews, and this decision can therefore be made prior to coding and category development. In a focus group study of HIV perceptions in Ghana, Ganle (2016) used the notion of saturation to determine when each focus group discussion should terminate. Such a decision would seem, however, to relate to only a very preliminary stage of analysis and is likely to be driven by only a rudimentary sense of any emergent theory. A similar point can be made in relation to Hancock el al.’s (2016) study of male nurses’ views on selecting a nursing speciality. They talk of logging each instance in which their focus group participants ‘discussed a theme’, with saturation then judged in relation to the number of times themes were discussed. Though not elaborated upon, this appears to imply a very narrow definition of a theme as something that can be somehow ‘observed’ during the course of a focus group. However, interpretations at this stage regarding what might constitute a theme, before even beginning to consider whether identified themes are saturated, will be superficial at best. Moreover, conclusions reached at this stage may not be particularly informative as regards subsequent theory development—pieces of data that appear to be very similar when first considered may be found to exemplify different theoretical constructs on detailed analysis, and correspondingly, data that are empirically dissimilar may turn out to have much in common theoretically. Judgments at this stage will also relate to a framework of themes and categories that is theoretically immature, and that may be subject to considerable modification; for example, the changes that may occur during the successive stages of open, selective and theoretical coding in grounded theory (Glaser 1978).

With regard to the second model identified, inductive thematic saturation, the fact that the focus is more explicitly on reaching saturation at the level of analysis—i.e. in relation to the (non-)emergence of new codes or themes—might suggest it will be achieved at a later stage than in data saturation approaches (notwithstanding the concurrent nature of data-collection and analysis in many qualitative approaches). However, focusing on the emergence or otherwise of codes rather than on their theoretical development still points us towards saturation being achieved at a relatively early stage. Hennink et al. (2017) highlight this in a study on patient retention in HIV care, in which they found that saturation of codes was achieved at an earlier point than saturation of the ‘dimensions, nuances, or insights’ related to codes. Hennink et al. argue that an approach to saturation relying only on the number of codes ‘misses the point of saturation’ (2017: p. 15) owing to a lack of understanding of the ‘meaning’ of these codes.

In contrast to data saturation and inductive thematic saturation, the first model of saturation considered, theoretical saturation—as based on the grounded theory notion of determining when the properties of theoretical categories are adequately developed—indicates that the process of analysis is at a more advanced stage and at a higher level of theoretical generality. Accordingly, Zhao and Davey (2015: p. 1178) refer to a form of saturation determined by ‘theoretical completeness’ and ceased sampling ‘when dimensions and gaps of each category of the grounded theory had been explicated,’ and Bowen (2008) gives a detailed account of how evidence of saturation emerged at the level of thematic categories and the broader process of theory construction.

### Saturation as event or process

A key issue underlying the identification of saturation is the extent to which it is viewed as an event or a process. Commonly, saturation is referred to as a ‘point’ (e.g. Otmar et al. 2011; Jassim and Whitford 2014; Kazley et al. 2015), suggesting that it should be thought of as a discrete event that can be recognized as such by the analyst. Strauss and Corbin (1998: p. 136), however, talk about saturation as a ‘matter of degree’, arguing that there will always be the potential for ‘the “new” to emerge’. They suggest that saturation should be more concerned with reaching the point where further data collection becomes ‘counter-productive’, and where the ‘new’ does not necessarily add anything to the overall story or theory. Mason (2010) makes a similar argument, talking of the point at which there are ‘diminishing returns’ from further data-collection, and a number of researchers seem to take this more incremental approach to saturation. Aiken et al. (2015: p. 154), for example, refer in their interview study of unintended pregnancy to being ‘confident of having achieved or at least closely approached thematic saturation.’ Nelson (2016), echoing Dey’s (1999) earlier view, argues that the term ‘saturation’ is itself problematic, as it intuitively lends itself to thinking in terms of a fixed point and a sense of ‘completeness’. He thus argues that ‘conceptual depth’ may be a more appropriate term—at least from a grounded theory perspective—whereby the researcher considers whether sufficient depth of understanding has been achieved in relation to emergent theoretical categories.

On this incremental reading of saturation, the analysis does not suddenly become ‘rich’ or ‘insightful’ after that one additional interview, but presumably becomes rich*er* or *more* insightful. The question will then be ‘how much saturation is enough?’, rather than ‘has saturation occurred?’^7^ This is a less straightforward question, but one that much better highlights the fact that this can only be a matter of the analyst’s decision—saturation is an ongoing, cumulative judgment that one makes, and perhaps never completes,^8^ rather than something that can be pinpointed at a specific juncture.

### Uncertainty and equivocation

A desire to identify a specific point in time at which saturation is achieved seems often to give rise to a degree of uncertainty or equivocation. In a number of studies, saturation is claimed, but further data collection takes place in an apparent attempt to ‘confirm’ (Jassim and Whitford 2014: p. 191; Forsberg et al. 2000: p. 328) or ‘validate’ (Vandecasteele et al. 2015: p. 2789) this claim; for example:

After the 10th interview, there were no new themes generated from the interviews. Therefore, it was deemed that the data collection had reached a saturation point. We continued data collection for two more interviews to ensure and confirm that there are no new themes emerging (Jassim and Whitford (2014: pp. 190–191).

Furthermore, a reluctance to rely on evidence of saturation sometimes indicates that saturation is being used in at best an unclear, or at worst an inconsistent or incoherent, fashion. For example, Hill et al. (2014: p. 2), whilst espousing the principle of saturation, seem not fully to trust it:

Saturation was monitored continuously throughout recruitment. For completeness we chose to fully recruit to all participant groups to reduce the chance of missed themes.

Similarly, Jackson et al. (2000: p. 1406) claim that saturation had been established, but then appear to retreat somewhat from this conclusion:

Following analysis of eight sets of data, data saturation was established… however, two additional participants were recruited to ensure data saturation was achieved.

Constantinou et al. (2017) propose that, given the potential for uncertainty about the point at which saturation is reached, attention should focus more on providing evidence that saturation has been reached, than on concerns about the point at which this occurred. Thus, rather curiously, they propose that it ‘does not hurt to include all interviews from the initial sampling’ (2017: p. 13). This view is inherently problematic, however, as not only does it imply that saturation is a retrospective consideration following the completion of data collection, rather than as guiding ongoing sampling decisions, but one could also argue that saturation loses its relevance if all data are included regardless of whether or not they contribute further insights or add to conceptual understanding. This approach appears to indicate a preoccupation with having enough data to show evidence of saturation, i.e. not too few interviews, rather than saturation aiding decisions about the adequacy of the sample.

Whilst the above suggests ambivalence towards assessing the point at which saturation is achieved, others report having made the conscious decision to continue sampling beyond saturation, appearing to seek additional objective evidence to bolster their sampling decisions. For instance, in investigating staff and patient views on a stroke unit, Tutton et al. (2012: p. 2063) talk of how, despite having achieved saturation, ‘increased observation may have increased the degree of immersion in the lives of those on the unit’, whilst Naegeli et al. (2013: p. 3) look to gain ‘more in-depth understanding… beyond the saturation point’. Similar points are made by Kennedy et al. (2012: p. 859), who talk of looking for ‘novel aspects’ after the achievement of saturation, and Poletti et al. (2007: p. 511), who propose the need to ‘fill gaps in the data’ following saturation. These examples suggest a view that there is something of theoretical importance that is not captured by saturation, though it is unclear from the explanations given as to exactly what this is.^9^

Another indication of an ambivalent view taken on saturation is suggested by Mason’s (2010) observation that sample sizes in studies based on interviews are commonly multiples of ten. This suggests that, in practice, rules of thumb or other a priori guidelines are commonly used in preference to an adaptive approach such as saturation. Quite frequently, studies that adopt the criterion of saturation propose at the same time a prior sample size (e.g. McNulty et al. 2015; Long-Sutehall et al. 2011). In a similar way, Niccolai et al. (2016) sought saturation during their analysis, but also state (p. 843) that:

An a priori sample size of 30 to 40 was selected based on recommendations for qualitative studies of this nature… and the anticipated complexity and desired level of depth for our research questions.

Fusch and Ness (2015: p. 1409) appear to endorse this somewhat inconsistent approach when advocating that the researcher should choose a sample size that has ‘the best opportunity for the researcher to reach data saturation’.^10^

This tentative and equivocal commitment to saturation may reflect a practical response to the demands of funding bodies and ethics committees for a clear statement of sample size prior to starting a study (O’Reilly and Parker 2013)—perceived obligations that, in practice, may be given priority over methodological considerations. However, it may also arise from the specific but somewhat uncertain logic that underlies saturation. Determining that further data collection or analysis is unnecessary on the basis of what has been concluded from data gathered hitherto is essentially a statement about the unobserved (what would have happened if the process of data collection and/or analysis had proceeded) based on the observed (the data collection and/or analysis that has taken place hitherto). Furthermore, if saturation is used in relation to negative case analysis in grounded theory (i.e. sources of data that may question or disconfirm aspects of the emergent theory) the logic becomes more tenuous—a statement about the unobserved based on the unobserved.^11^ In either case, an uncertain predictive claim is made about the nature of data yet to be collected, and furthermore a claim that could only be tested if the decision to halt data collection were to be overturned. Additionally, the underlying reasoning makes specific assumptions about the way in which the analysis will generate theory, and the earlier in the process of theory development that this occurs the less warranted such assumptions may be. Accordingly, researchers who confidently propose saturation as a criterion for sampling at the outset of a study may become less certain as to how it should be operationalized once the study is in progress, and may therefore be reluctant to abide by it.

## Conclusion

This paper has offered a critical reflection on the concept of saturation and its use in qualitative research, contributing to the small body of literature that has examined the complexities of the concept and its underlying assumptions. Drawing on recent examples of its use, saturation has been discussed in relation to three key sets of questions: What? Where and why? When and how?

Extending previous literature that has highlighted the variability in the use of saturation (O’Reilly and Parker 2013; Walker 2012), we have scrutinized the different ways in which it has been operationalized in the research literature, identifying four models of saturation, each of which appears to make different core assumptions about what saturation is, and about what exactly is being saturated. These have been labelled as: theoretical saturation, inductive thematic saturation, a priori thematic saturation, and data saturation. Moving forward, the identification and recognition of these different models of saturation may aid qualitative researchers in untangling some of the inconsistencies and contradictions that characterize its use.

Saturation’s apparent position as a ‘gold standard’ in assessing quality and its near universal application in qualitative research have been previously questioned (Guest et al. 2006; O’Reilly and Parker 2013; Malterud et al. 2016). Similarly, doubts have been raised regarding its common adoption as a *sole* criterion of the adequacy of data collection and analysis (Charmaz 2005), or of the adequacy of theory development: ‘Elegance, precision, coherence, and clarity are traditional criteria for evaluating theory, somewhat swamped by the metaphorical emphasis on saturation’ (Dey 2007: p. 186). On the basis of such critiques, we have examined how saturation might be considered in relation to different theoretical and analytical approaches. Whilst we concur with the argument that saturation should not be afforded unquestioned status, polarization of saturation as either applicable or non-applicable to different approaches, as has been suggested (Walker 2012), may be too simplistic. Instead we propose that saturation has differing relevance, and a different meaning, depending on the role of theory, the analytic approach adopted, and so forth, and thus may usefully serve different purposes for different types of research—purposes that need to be clearly articulated by the researcher.

Whilst arguing for flexibility in terms of the purpose and use of saturation, we also suggest that there must be some limit to this range of purposes. Some of the ways in which saturation has been operationalized, we would suggest, risk stretching or diluting its meaning to the point where it becomes too widely encompassing, thereby undermining its coherence and utility.

When and how saturation may be judged to have been reached will differ depending on the type of study, as well as assumptions about whether it represents a distinct event or an ongoing process. The view of saturation as an event has been problematized by others (Strauss and Corbin 1998; Dey 1999; Nelson 2016), and we have explored the implications of conceptualizing saturation in this way, arguing that it appears to give rise to a degree of uncertainty and equivocation, in part driven by the uncertain logic of the concept itself—as a statement about the unobserved based on the observed. This uncertainty appears to give rise to inconsistencies and contradictions in its use, which we would argue could be resolved, at least in part, if saturation were to be considered as a matter of degree, rather than simply as something either attained or unattained. However, whilst considering saturation in incremental terms may increase researchers’ confidence in making claims to it, we suggest it is only through due consideration of the specific purpose for which saturation is being used, and what one is hoping to saturate, that the uncertainty around the concept can be resolved.

In highlighting and examining these areas of complexity, this paper has extended previous discussions of saturation in the literature. Whilst consideration of the concept has led some commentators to argue for the need for qualitative researchers to provide a more thorough and transparent reporting of how they achieved saturation in their research, thus allowing readers to assess the validity of this claim (Bowen 2008; Francis et al. 2010), our arguments go beyond this. We contend that there is a need not only for more transparent reporting, but also for a more thorough re-evaluation of how saturation is conceptualized and operationalized, including recognition of potential inconsistencies and contradictions in the use of the concept—this re-evaluation can be guided through attending to the four approaches we have identified and their implications for the purposes and uses of saturation. This may lead to a more consistent use of saturation, not in terms of its always being used in the same way, but in relation to consistency between the theoretical position and analytic framework adopted, allowing saturation to be used in such a way as to best meet the aims and objectives of the research. It is through consideration of such complexities in the context of specific approaches that saturation can have most value, enabling it to move away from its increasingly elevated yet uneasy position as a taken-for-granted convention of qualitative research.

## Footnotes

1 Although primarily employed in primary research, principles of saturation have also been applied to qualitative synthesis (Garrett et al. 2012; Lipworth et al. 2013). However, our focus here is on its use in primary studies.

2 These authors proceed to make the more extreme claim that saturation ‘is important in any study, whether quantitative, qualitative, or mixed methods’ (Fusch and Ness 2015: p. 1411).

3 It should be noted that Birks and Mills (2015) also state that, as part of theoretical saturation, ‘Categories are clearly articulated with sharply defined and dimensionalized properties’, suggesting a somewhat broader view of saturation, in which the nature of emerging themes is important, rather than just the fact of their (non)emergence.

4 This evokes Glaser’s criticism of Strauss’s approach to sampling, which he regards as conventional, rather than theoretical, sampling: ‘In conventional sampling the analyst questions, guesses and uses experience to go where he thinks he will have the data to test his hypotheses and find the theory that he has preconceived. Discovery to Strauss does not mean induction and emergence, it means finding his theory in data so that it can be tested’ (Glaser 1992: p. 103).

5 Charmaz (2008, 2014) is critical of the extension of the notion of saturation beyond the context of grounded theory, and in particular of its extension into what we here refer to as data saturation.

6 Few authors draw an explicit distinction between data and theoretical saturation—among the exceptions are Bowen (2008), Sandelowski (2008), O’Reilly and Parker (2013), and Hennink et al. (2017).

7 Hence, Dey (1999: p. 117) suggests the term ‘sufficiency’ in preference to ‘saturation’.

8 This reflects Glaser and Strauss’s (1967: p. 40) view of theory generation: ‘one is constantly alert to emergent perspectives that will change and help develop his theory. These perspectives can easily occur even on the final day of study or even when the manuscript is reviewed in page proof; so the published word is not the final one, but only a pause in the never-ending process of generating theory’.

9 On occasions, a reason for going beyond saturation appears to be ethical rather than methodological. Despite reaching saturation, France et al. (2008: p. 22) note that owing to their ‘commitment to and respect for all the women who wanted to participate in the study, data collection did not end until all had been interviewed.’ Similarly, Kennedy et al. (2012: p. 858) report that they exceeded saturation as this appeared to be ‘more ethical than purposefully choosing individuals to re-interview, or only interviewing until saturation’.

10 Bloor and Wood (2006: p. 165) suggest that this tendency may stem from researchers feeling obliged to abide by sample sizes previously declared to funding bodies or ethics committees, whilst making claims to saturation in order to retain a sense of methodological credibility. Some authors—e.g. Guest et al. (2006), Francis et al. (2010), Hennink et al. (2017)—have attempted for formulate procedures whereby the specific number of participants required to achieve saturation is calculated in advance.

11 The first logic is counter-inductive—future non-occurrences of data, codes or theoretical insights are posited on the basis of prior occurrences. In relation to negative case analysis, however, the logic becomes inductive—future non-occurrences are posited on the basis of prior non-occurrences.
